# Desensitized D2 autoreceptors are resistant to trafficking

**DOI:** 10.1038/s41598-017-04728-z

**Published:** 2017-06-29

**Authors:** Brooks G. Robinson, James R. Bunzow, Jonathan B. Grimm, Luke D. Lavis, Joshua T. Dudman, Jennifer Brown, Kim A. Neve, John T. Williams

**Affiliations:** 1 The Vollum Institute, Oregon Health and Science University, 3181S.W. Sam Jackson Pk. Rd., Portland, OR 97239 USA; 2 Howard Hughes Medical Institute, Janelia Research Campus, 19700 Helix Dr., Ashburn, VA 20147 USA; 3 Research Service, VA Portland Health Care System; and Department of Behavioral Neuroscience, Oregon Health and Science University, 3181S.W. Sam Jackson Pk. Rd., Portland, OR 97239 USA

## Abstract

Dendritic release of dopamine activates dopamine D2 autoreceptors, which are inhibitory G protein-coupled receptors (GPCRs), to decrease the excitability of dopamine neurons. This study used tagged D2 receptors to identify the localization and distribution of these receptors in living midbrain dopamine neurons. GFP-tagged D2 receptors were found to be unevenly clustered on the soma and dendrites of dopamine neurons within the substantia nigra pars compacta (SNc). Physiological signaling and desensitization of the tagged receptors were not different from wild type receptors. Unexpectedly, upon desensitization the tagged D2 receptors were not internalized. When tagged D2 receptors were expressed in locus coeruleus neurons, a desensitizing protocol induced significant internalization. Likewise, when tagged µ-opioid receptors were expressed in dopamine neurons they too were internalized. The distribution and lack of agonist-induced internalization of D2 receptors on dopamine neurons indicate a purposefully regulated localization of these receptors.

## Introduction

Dopamine D2 receptors are crucial for many behavioral processes including movement and the response to rewarding stimuli^1–3^ and thus are also involved in a number of disease states including Parkinson’s disease, drug addiction, and schizophrenia. The regulation and adaptation of D2 receptor signaling is important for understanding basic behaviors, the etiology of many diseases, and the treatment of those diseases. The mechanisms underlying adaptive processes such as desensitization and tolerance remain incompletely characterized for the D2 receptor.

D2 receptors are functionally diverse and serve both as heteroreceptors expressed on neurons in projection targets of dopamine neurons as well as autoreceptors expressed on dopamine neurons. Autoreceptors are expressed on axon terminals to regulate neurotransmitter release as well as on the somata and dendrites of dopamine neurons to regulate neuronal excitability. Within the ventral tegmental area (VTA) and substantia nigra pars compacta (SNc), dopamine neurons communicate via dendrodendritic neurotransmission. Structural studies first identified presynaptic dendrites of unknown origin in the SNc in 1973^4^. Catecholamines were prominently detected within dendritic varicosities of dopamine neurons shortly thereafter^5^ leading to the verification of dendrodendritic synapses between dopamine neurons in the SNc^6, 7^. Altered dopamine neuron firing in response to exogenous dopamine^8^ indicated that dopamine neurons could self regulate^9^. Dendritic dopamine release has since been directly measured using several methods^10–14^ and the target of dopamine is the D2 autoreceptor^15–18^. Activation of the D2 receptor by dopamine results in a hyperpolarizing potassium conductance through an inwardly rectifying channel^17^that is dependent on G protein signaling^19, 20^.

Thus, within midbrain nuclei, dopamine neuron excitability is regulated through somatodendritic release of dopamine acting on G protein-coupled D2 receptors^21^. An unresolved question in the field is the method by which D2 receptor-dependent dopamine transmission occurs in the midbrain. Are there synapses containing paired release sites and receptors or is volume transmission the primary mode of communication between midbrain neurons? The presence of transient, quantal synaptic events involving the D2 receptor^14, 22, 23^ is at odds with the anatomical data indicating that D2 receptors primarily reside at distances from dopamine release sites requiring significant diffusion of the neurotransmitter^24–26^.

In this study, a novel mouse model with a superecliptic pHluorin (GFP) knocked-in to the N-terminus of the endogenous D2 receptor (henceforth GFP-D2; Supplementary Figure 1) was used to examine the distribution and functional consequences of D2 autoreceptor activation in live neurons from the SNc. The tagged receptor functions normally as measured by the ability to activate a GIRK conductance. The results also show that the receptors are unevenly distributed into clusters or puncta on the cell membrane. Furthermore, D2 receptors in dopamine neurons do not readily internalize upon agonist exposure.

## Results

### A punctate distribution of the GFP-D2 receptors

**Figure 1:**
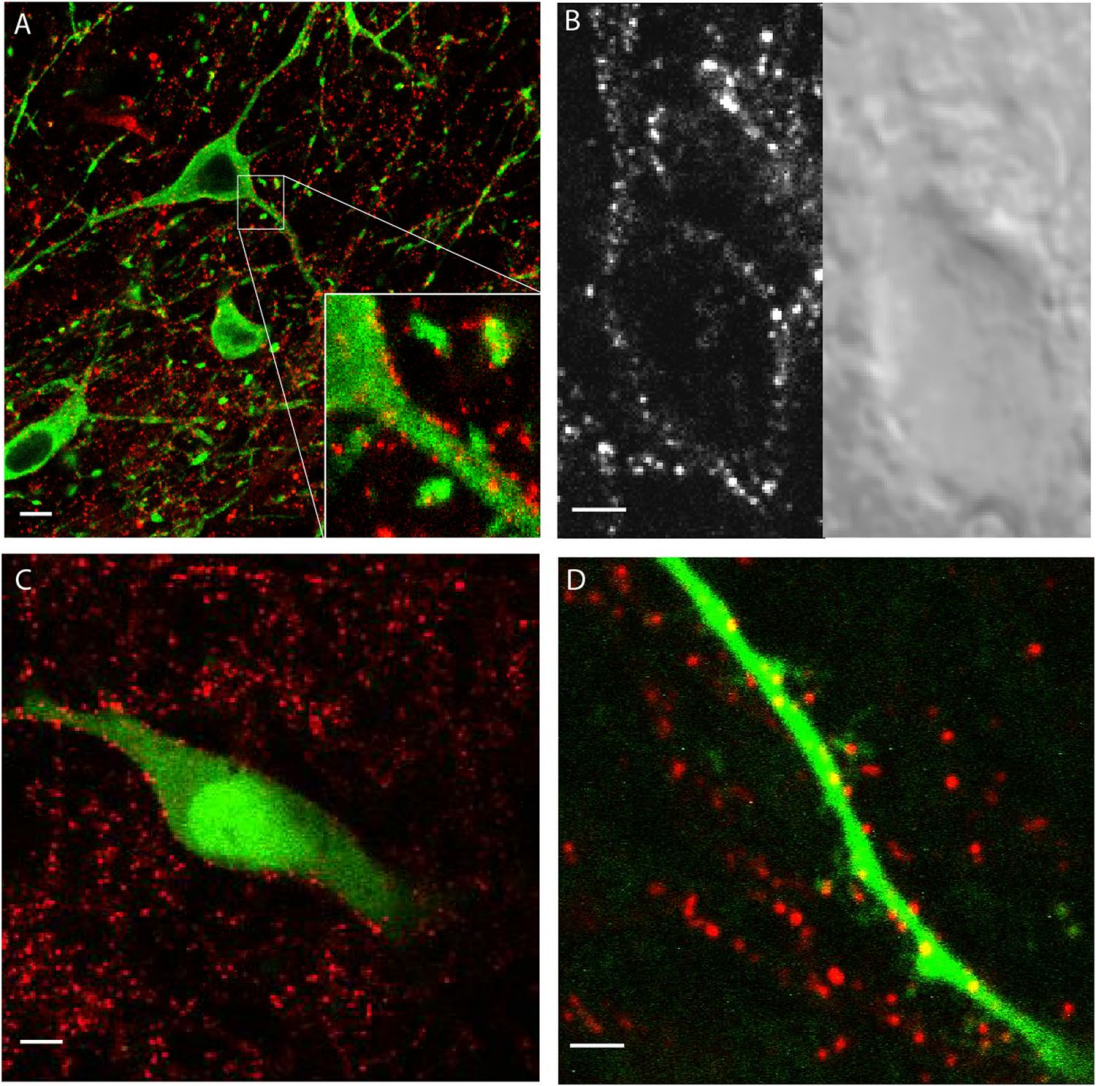
Visualizing the GFP-D2 receptor. (**A**) Confocal image of a slice from a GFP-D2 animal. Slice incubated in anti-GFP-AF594 (red) to amplify the signal on the D2 receptor prior to fixation. Tyrosine hydroxylase (TH) stained in green. (**B**) Simultaneously acquired 2-photon (left) and DIC (right) images of a live neuron from a GFP-D2 acute section. Slice was incubated in anti-GFP-AF594 prior to imaging. (**C**) 2-photon imaging of a live neuron filled with AF488. Slice was incubated in anti-GFP-AF594 prior to imaging. (**D**) 2-photon image zoomed in on a distal dendrite from a neuron filled with AF488. Receptors amplified with anti-GFP-AF594 (red) seen on dendritic shaft and spine-like structures. A-C scale bars 5 µm, D scale bar 1 µm.

The plasma membrane bound GFP-D2 receptors were examined in living brain slices using an excitation wavelength of 810 nm in a custom-built 2-photon microscope and in fixed slices with confocal microscopy. The superecliptic pHluorin version of GFP was used to tag the D2 receptor because only membrane-bound receptors would be visible (the higher pH inside cells quenches the GFP). However, the intensity of the superecliptic pHluorin GFP is less than GFP and without antibody amplification was below the limit of detection in both fixed and living slice preparations. Therefore in each case, living slices were incubated with an anti-GFP antibody conjugated to Alexa Fluor 594 (AF594) for 30 min. Slices used for confocal microscopy were fixed and tyrosine hydroxylase was immunostained (Fig. 1A). For imaging of live cells, the slices were imaged directly following incubation in the antibody (Fig. 1B–D).

In each case, the labeled GFP-D2 receptors lined the membrane of the cell body and primary dendrites with a decidedly punctate distribution. This punctate pattern continued from the cell soma to distal dendrites and, in some cases, was seen on spines as illustrated in Fig. 1D. This uneven distribution of receptors is unlike other GPCRs shown to be evenly distributed along the plasma membrane^27^. The uneven distribution of D2 receptors was also observed using a transgenic animal where the tyrosine hydroxylase promotor drives the expression of a D2 receptor modified with a FLAG epitope at the N-terminal^22, 28^. Thus, the punctate distribution was observed in two animal models that expressed distinctly tagged receptors.

One concern was the potential that the uneven distribution of fluorescence was the result of antibody-induced alterations in the distribution and/or clustering of receptors. Experiments using an anti-GFP nanobody were used to address this concern. Nanobodies do not crosslink as they are univalent and only recognize a single site on the GFP. The molecular weight is about 1/10^th^ that of an IgG, and each nanobody was conjugated with a single fluorophore (AF594) so the signal is not amplified as it can be with IgGs that often have multiple fluorophores bound. Thus, the penetration of these fluorescent antibodies into the living brain slice is significantly greater than labeled anti-GFP IgG antibodies. The GFP-D2 receptor distribution using this method was similar to that obtained with the IgG antibody, suggesting that the antibody being bound does not affect the localization and therefore did not account for the punctate distribution of GFP-D2 receptors (Supplementary Figure 2A). Because this nanobody was previously untested, brain slices from WT animals were incubated in the nanobody to determine specificity. There was no detectable nanobody binding to live cells in the SNc (Supplementary Figure 2B) indicating that the fluorescent signal from the nanobody was specific to the GFP antigen. The nanobody did occasionally accumulate in dead cells (from both GFP-D2 and WT slices), and thus was not used in experiments involving receptor internalization.

### Tagged D2Rs function like wild type receptors

**Figure 2:**
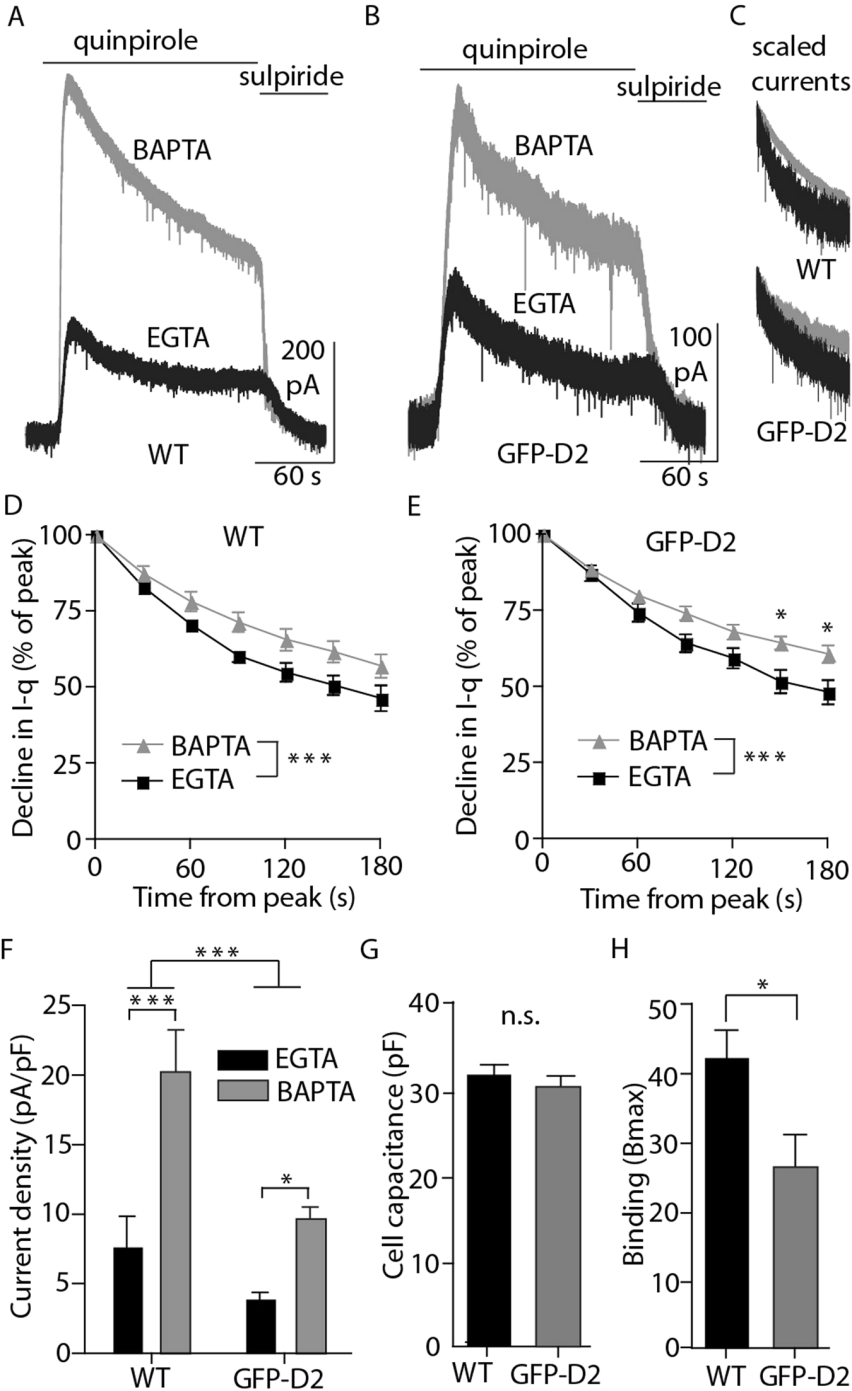
Knocked-in D2 receptor functions in dopamine neurons, but is expressed at levels below the wild type. (**A,B**) Representative whole-cell voltage clamp recordings of dopamine neurons from wild type and GFP-D2 acute slices during the bath application of the D2 agonist quinpirole (10 µM) followed by the antagonist sulpiride (600 nM). (**C**) Example BAPTA and EGTA currents from wild type (top) and GFP-D2 (bottom) scaled and peak aligned. (**D**) In wild type, the quinpirole-induced current declines significantly more from peak value when EGTA internal is used compared to BAPTA (n = 5 and 7, two-way ANOVA followed by Bonferroni). (**E**) In the GFP-D2, the quinpirole-induced current also declines significantly more from peak value when EGTA internal is used compared to BAPTA (n = 7 and 10, two-way ANOVA followed by Bonferroni). (**F**) In both wild type and GFP-D2, the quinpirole-induced current density was larger using BAPTA internal compared with EGTA internal. As a group, wild type current density was larger than that for GFP-D2 (n = 5–10, two-way ANOVA followed by Bonferroni). (**G**) The average cell capacitance did not differ between the wild type and GFP-D2 groups (n = 21 and 29, unpaired t test). (**H**) The density of D2 receptors from midbrain samples as measured by the binding of [^3^H]YM-09151-2, was significantly greater in wild type compared to GFP-D2 (n = 9 each, unpaired t test). *p < 0.05, ***p < 0.001.

It was vital to determine whether the tagged D2 receptor functioned normally. Whole cell voltage clamp recordings were made from dopamine neurons in the SNc from wild type (WT) and GFP-D2 knock-in mice. Application of quinpirole (10 µM) in slices from GFP-D2 animals induced an outward G protein-coupled inwardly rectifying potassium (GIRK)-mediated current that closely resembled that from slices take from WT animals (Fig. 2A,B). In WT animals, D2-GIRK currents have been found to be sensitive to internal calcium buffering. To determine if this characteristic is preserved in the GFP-D2 animal, D2-GIRK currents in response to quinpirole (10 µM) were measured using a high buffering 10 mM BAPTA internal solution and a low buffering 0.1 mM EGTA internal solution. As previously shown, the desensitization (measured as a reduction in the current amplitude during agonist exposure) of D2-GIRK currents was different between the BAPTA and EGTA internals in recordings from WT animals (examples of scaled, peak aligned currents shown in Fig. 2C). When scaled and peak-aligned, the quinpirole induced D2-GIRK current (I-q) declined to a greater extent when the low calcium buffering EGTA internal was used (Fig. 2D). Recordings from cells in GFP-D2 slices showed the same calcium sensitivity of D2-GIRK currents as WT (Fig. 2E). Additionally, the current amplitudes differ between the two internal solutions with those using EGTA being significantly smaller than BAPTA (Fig. 2F). The same pattern was present in recordings from GFP-D2 slices, however the maximal current amplitude produced in cells from GFP-D2 slices was significantly smaller than seen in WT (Fig. 2F). Current amplitude is correlated with both receptor density and cell size. The cell capacitance (an electrophysiological measure of cell size) was found to be the same in WT and GFP-D2 cells (Fig. 2G). Furthermore, the D2 receptor density in a midbrain membrane preparation, determined by radioligand binding, was reduced in the samples from GFP-D2 animals compared to WT (Fig. 2H). Therefore, the reduced D2 receptor-mediated current density in the GFP-D2 animal compared to WT is probably because of decreased expression of the GFP-modified receptor.

**Figure 3:**
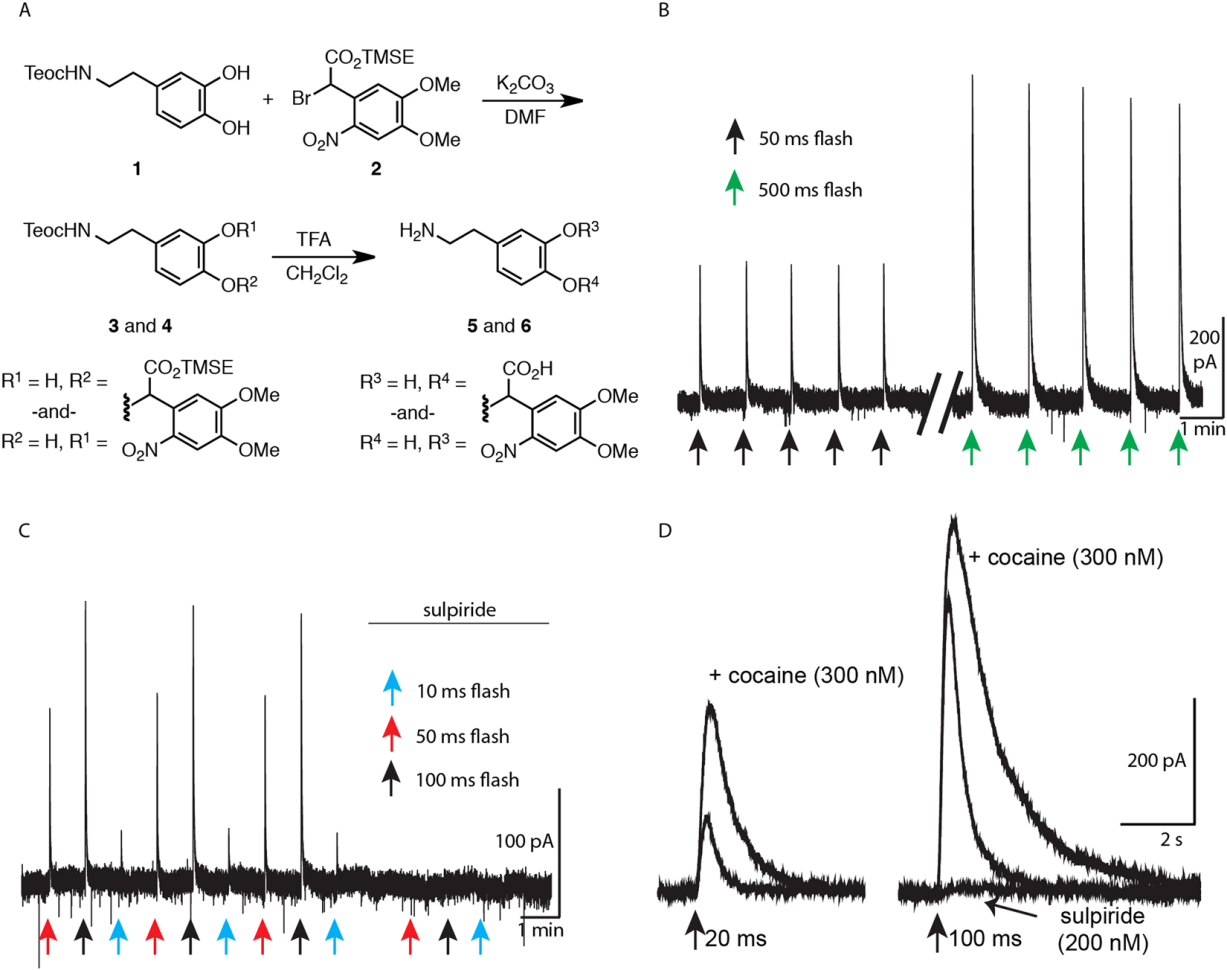
Synthesis and photolysis of caged dopamine. (**A**) Synthetic rout to caged dopamine (CNV-dopamine, 5 and 6). See methods for detailed explanation. (**B**) The caged dopamine compound was photolysed with LED flashes of 365 nm. When flashes were conducted with a 60 second interval, there was little to no rundown of the resultant D2-GIRK current with flash lengths up to 100 ms. Even with longer 500 ms flashes, only a modest decrease in current was observe. This was likely due to receptor desensitization rather than compound rundown. (**C**) All flash-induced currents were completely abolished by the addition of sulpiride to the caged dopamine reperfusion solution indicating the dopamine being uncaged acted solely on the D2 receptor. (**D**) Application of the dopamine transporter (DAT) blocker cocaine increases the amplitude and prolongs the signaling of the flash currents indicating that the uncaged dopamine is taken up through the DAT.

The signaling kinetics of the WT and tagged receptors were examined using a novel photoactivatable (caged) dopamine: carboxynitroveratryl-dopamine (CNV-dopamine; Fig. 3A). Replacement of the carboxynitrobenzyl (CNB) photolabile group from a previously described photoactivatable CNB-dopamine^29^ with the CNV cage shifted the absorption maxima of the cage to longer wavelenths, allowing efficient activation with 365 nm light. The compound was dissolved in ACSF to achieve a concentration of 100 µM and recirculated over the slice. Full field LED (365 nm) flashes with a 60 s inter-flash interval were used and responses were stable for the length of the recording (Fig. 3B). The D2 receptor antagonist sulpiride abolished all flash-induced current (Fig. 3C) and the cellular responses to the uncaged dopamine were regulated by uptake through the dopamine transporter (Fig. 3D).

**Figure 4:**
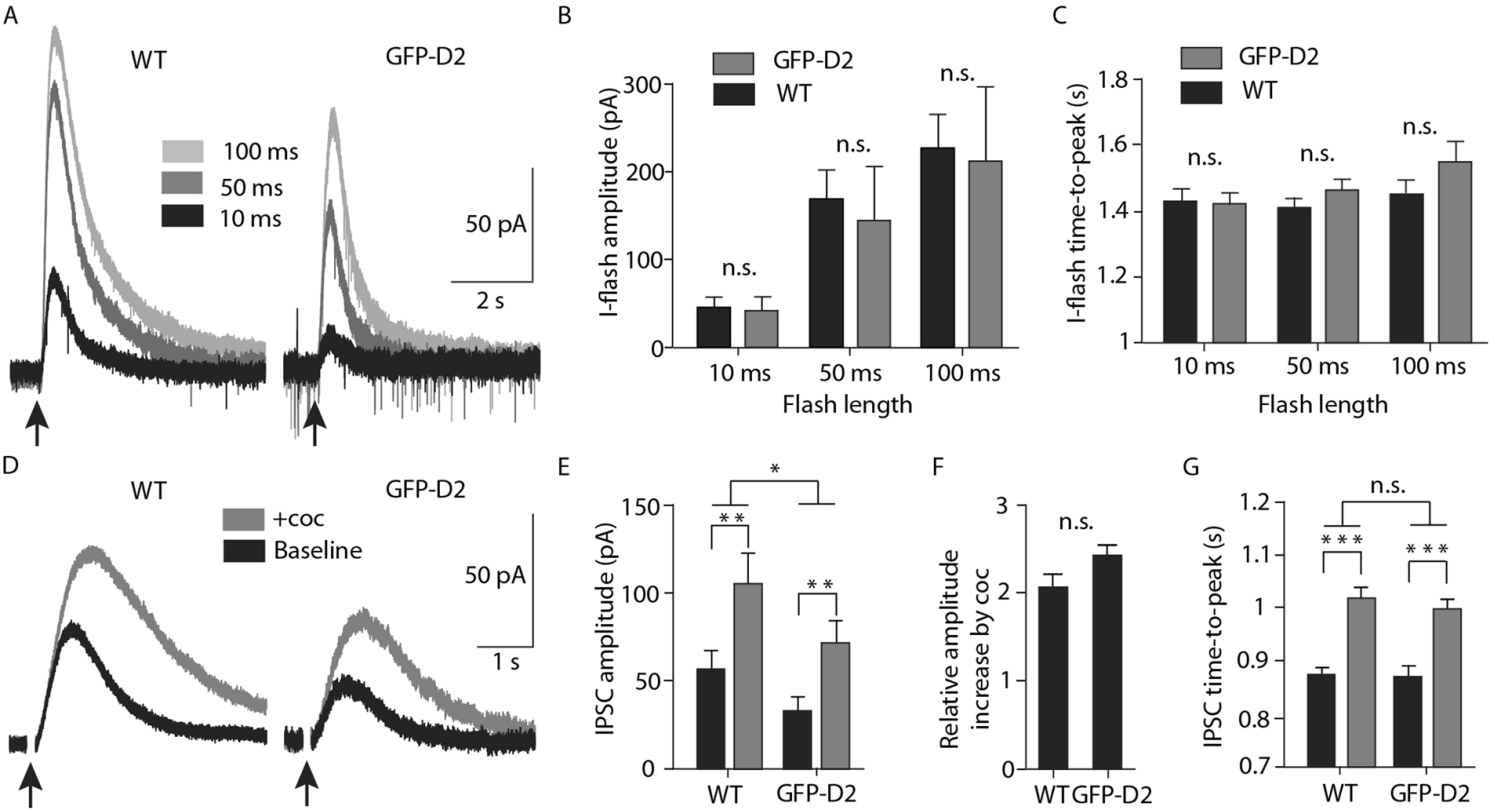
Signaling kinetics of the GFP-D2 resemble that of wild type D2 receptor. (**A**) Representative traces following the full-field LED uncaging (10, 50, 100 ms flashes) of dopamine onto wild type and GFP-D2 neurons. (**B**) The amplitude following dopamine uncaging did not differ between wild type and GFP-D2 at any of the flash lengths (n = 7 and 6, two-way ANOVA followed by Bonferroni). (**C**) The time-to-peak of the uncaged dopamine-induced currents were not significantly different between wild type and GFP-D2 (n = 7 and 6, two-way ANOVA followed by Bonferroni). (**D**) Representative traces of electrically stimulated D2-receptor mediated IPSCs from wild type and GFP-D2. Baseline IPSCs (black) are compared with those following the bath application of the DAT inhibitor cocaine (1 µM, gray). (**E,F**) Overall, the wild type IPSCs were larger than the GFP-D2. The amplitude of both the wild type and GFP-D2 IPSCs increased following cocaine application and the relative increase by cocaine was similar for wild type and GFP-D2 (n = 7 and 8, two-way ANOVA followed by Bonferroni, and unpaired t test). (**F**) The time-to-peak of wild type and GFP-D2 IPSCs both significantly increased following cocaine, and there was no overall difference between the wild type and GFP-D2 groups (n = 7 and 8, two-way ANOVA followed by Bonferroni). n.s. denotes not significant, *p < 0.05, **p < 0.01, ***p < 0.01.

For the comparison of WT and GFP-D2, 10, 50, and 100 ms flashes were used to uncage the dopamine (Fig. 4A). The peak amplitude (Fig. 4B) and the time-to-peak (Fig. 4C) of the flash currents (I-flash) were not significantly different between WT and GFP-D2 recordings. This is likely due to the large variability seen in peak amplitude. However, the WT trended towards being faster to peak than the GFP-D2 (p = 0.09).

Electrical stimulation within the SNc reliably evokes a D2-GIRK mediated inhibitory post-synaptic current (IPSC). Examples of electrically evoked IPSCs are shown in Fig. 4D. The kinetics of the IPSC (~1.5 s from start to finish) depend on a transiently high concentration of dopamine that is removed from the extracellular space through the dopamine transporter (DAT). Although the amplitude of IPSCs may vary considerably from cell to cell, there was little variability in kinetics. The IPSCs in GFP-D2 slices were significantly smaller than those from WT (Fig. 4E). However, the time-to-peak of the IPSCs did not differ between WT and GFP-D2. Inhibition of DAT using cocaine (1 µM) increased the amplitude (Fig. 4F) and time-to-peak (Fig. 4G) of IPSCs similarly in slices from both WT and GFP-D2 animals. These results indicate that, while the IPSCs were smaller in the GFP-D2, the receptors are localized in a cellular compartment that encounters a concentration of dopamine high enough to account for the rapid activation kinetics similar to that seen in WT.

### Antibody labeling does not alter the signaling or activation kinetics

To determine whether the antibody-bound receptors were functional, electrophysiological recordings were made from cells having antibody labeled D2 receptors (Supplementary Figure 3A). Application of either quinpirole (10 µM) or dopamine (100 µM) produced outward currents with acute desensitization equivalent to recordings from slices that were not incubated with antibody (Supplementary Figure 3B,C). The peak D2-GIRK amplitudes also did not change due to the antibody (Supplementary Figure 3D). Furthermore, the peak amplitude (Supplementary Figure 3E) and time-to-peak (Supplementary Figure 3F) of currents induced by the LED uncaging of dopamine (10, 50 or 100 ms flashes) did not differ between recordings from slices that were and were not incubated with antibody.

### Limited internalization of GFP-D2 receptors

**Figure 5:**
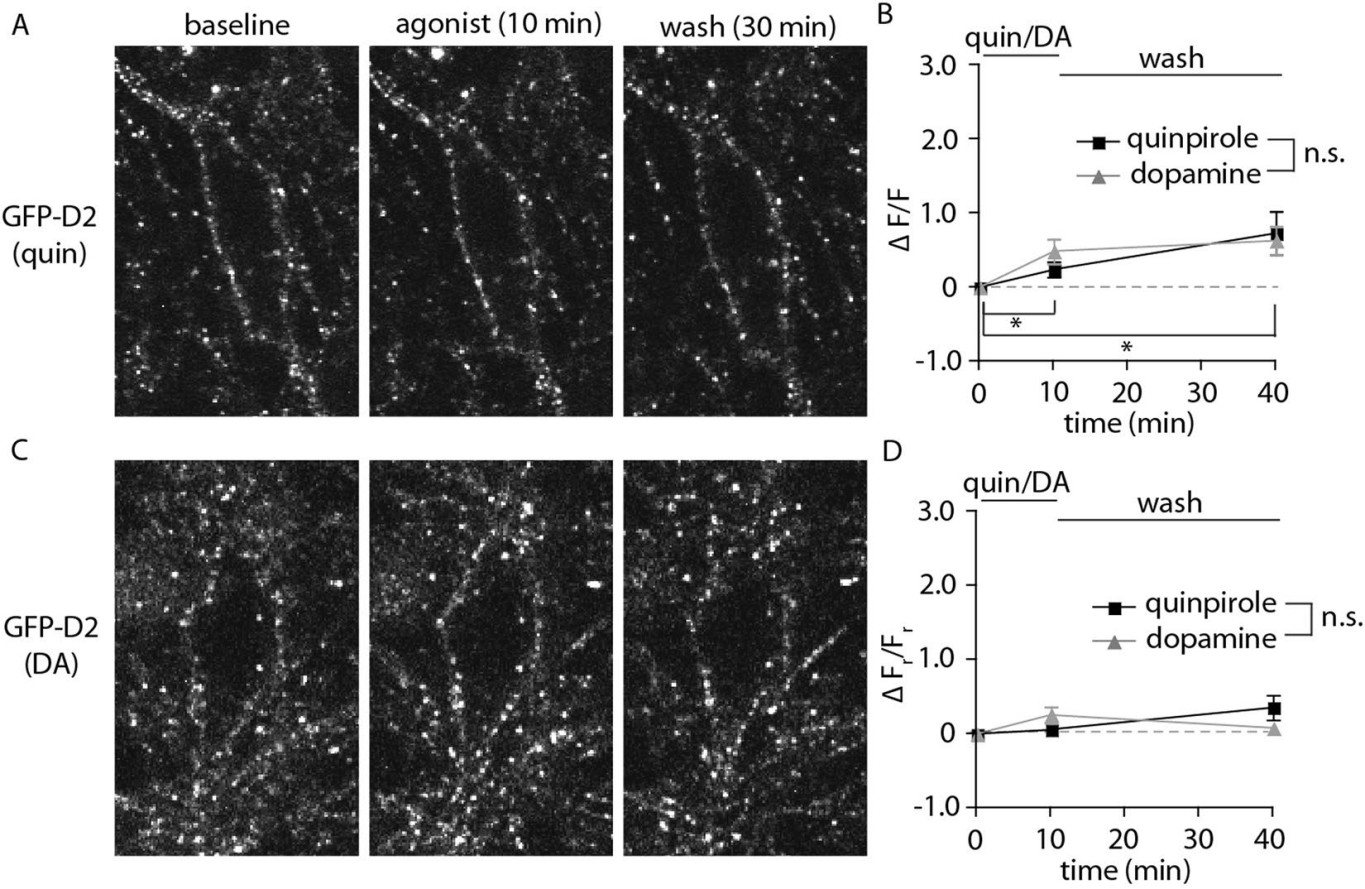
GFP-D2 receptors show limited internalization. (**A, C**) Representative 2-photon images of live neurons at baseline, following 10 minutes of agonist bath application (quinpirole 10 µM-A, dopamine 100 µM-C), and following 30 minutes of agonist-free wash. Slices were incubated in anti-GFP-AF594 to label GFP-D2 receptors prior to imaging. (**B**) The change in fluorescence inside the neuron (ΔF/F) increases modestly but significantly over baseline following the agonist application and wash. There was no difference between the agonists used (n = 6 for both, two-way ANOVA followed by Bonferroni). (**D**) The change in the ratio of intracellular fluorescence to membrane fluorescence (ΔF_r_/F_r_) did not change significantly from baseline following agonist application or wash (n = 6 for both, two-way ANOVA followed by Bonferroni). *p < 0.05.

GPCRs have canonically been thought to undergo arrestin-dependent internalization in response to agonists as a component of the mechanisms that underlie desensitization and recovery from desensitization. Using tagged D2 receptors, this hypothesis was directly tested in live neurons in brain slices with 2-photon laser microscopy. Neurons labeled with the antiGFP-AF594 antibody were imaged. At baseline a z-stack captured the entire depth of the cell body. A saturating concentration of agonist (quinpirole 10 µM or dopamine 100 µM) was applied for 10 minutes followed by the acquisition of a second image. Agonist was washed for 30 minutes and the cell was imaged again. Example neurons treated with quinpirole (Fig. 5A) or dopamine (Fig. 5C) show that very little internalization was detected. Most fluorescence remained on or very near the membrane for the duration of the experiment. Analysis was carried out in images that consisted of 3–5 merged slices (1 µm each) in the center of the neuron. The fluorescence on the membrane was measured by drawing around the outside of the cell membrane and subtracting the intracellular fluorescence, which was taken from the entire area just inside the membrane. Fluorescence values were normalized to the background of the image to account for non-specific changes through the duration of the experiment. Raw values of the change in fluorescence inside the cell from the baseline image (ΔF/F) indicate both agonists cause a small, but significant internalization of the D2 receptor (Fig. 5B). However, when the fluorescent (F) value is generated as the ratio of internal fluorescence to that on the membrane, there is no significant change following agonist exposure (Fig. 5D). This ratio (ΔF_r_/F_r_) controls for differences in the number of receptors initially bound by antibody (due to expression level or antibody penetration/depth of cell) and any antibody that washed during the experiment.

### Transgenic FLAG-D2 receptors

**Figure 6:**
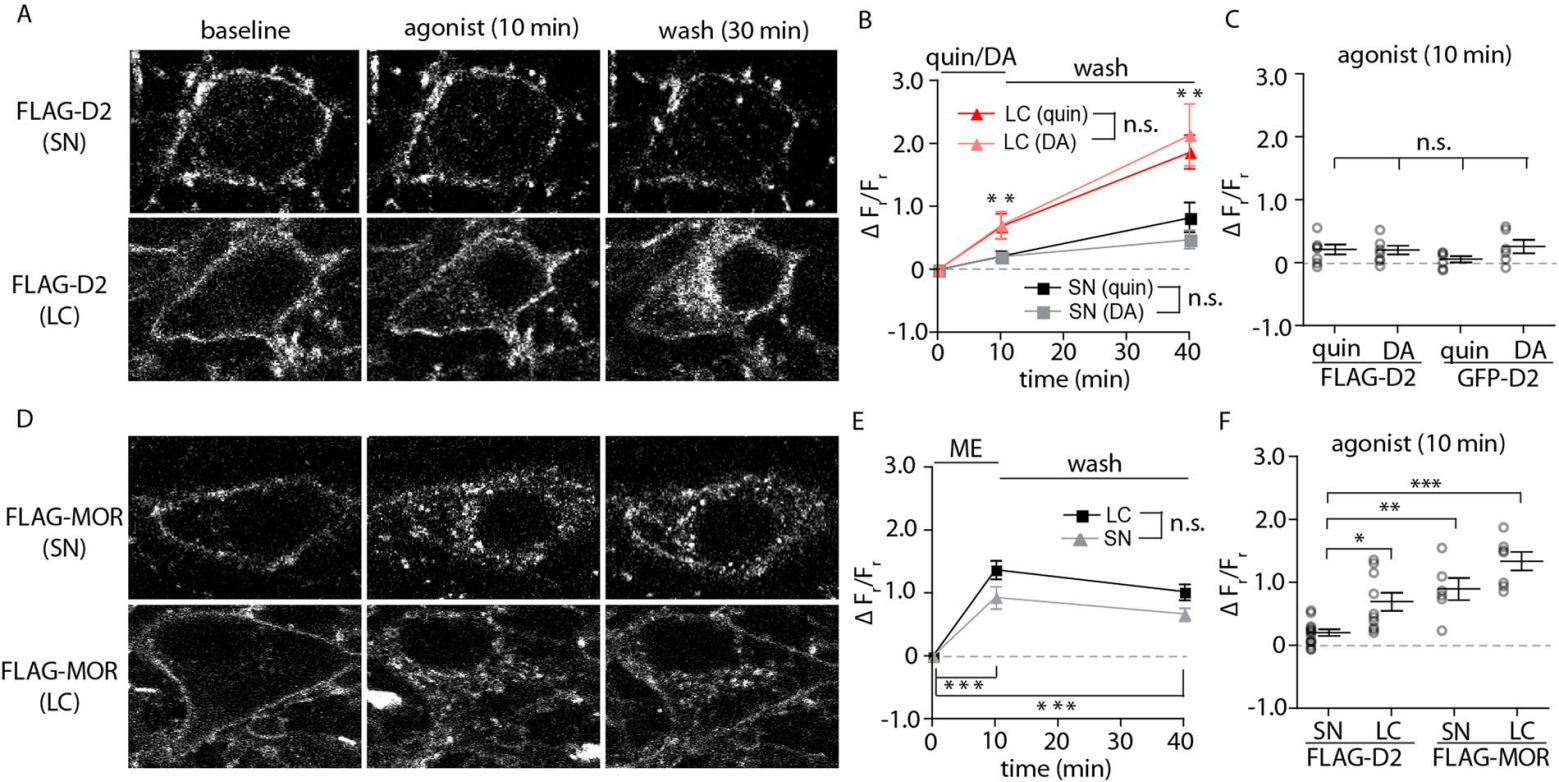
FLAG-tagged receptors reveal specificity of limited D2 receptor internalization. (**A**) Representative images of live neurons at baseline, following 10 minutes of agonist bath application (quinpirole 10 µM, dopamine 100 µM), and following 30 minutes of agonist-free wash. Slices were incubated in anti-FLAG M1-AF594 to label FLAG-tagged D2 receptors in the SN and LC prior to imaging. (**B**) FLAG D2 receptors in SN dopamine neurons show limited internalization following agonist exposure as measured by the change in the ratio of intracellular to membrane fluorescence (ΔF_r_/F_r_), only significant following the 30-min wash (not specified on graph). FLAG-D2 receptors in the LC show a significantly larger increase in the intracellular fluorescence relative to the membrane fluorescence (ΔF_r_/F_r_) when compared with FLAG-D2 receptors in SN neurons (n = 5–7, two-way ANOVA followed by Bonferroni). (**C**) The ΔF_r_/F_r_ following 10 minutes of agonist exposure was not significantly different between GFP-D2 and FLAG-D2 receptors for either quinpirole or dopamine (n = 5–7, two-way ANOVA followed by Bonferroni). (**D**) Representative images of live neurons at baseline, following 10 minutes of agonist bath application ([Met]^5^enkephalin ME, 30 µM), and following 30 minutes of agonist-free wash. Slices were incubated in anti-FLAG M1-AF594 to label FLAG-tagged µ-opioid receptors (MORs) in the SN and LC prior to imaging. (**F**) In both the SN and LC, FLAG-MORs show a sharp increase in ΔF_r_/F_r_ following agonist exposure. There was a slight decrease in ΔF_r_/F_r_ following the 30-minute wash period. (**F**) FLAG-D2 receptors in the LC and FLAG-MORs in both the SN and LC show significantly larger ΔF_r_/F_r_ following agonist application than FLAG-D2 receptors in the SN (n = 6–14, two-way ANOVA followed by Bonferroni). n.s. denotes not significant, *p < 0.05, **p < 0.01, ***p < 0.01.

The relative lack of internalization of GFP-D2 was a surprising result, especially with the appearance of normal signaling and desensitization particularly after treatment with antibody (Supplementary Figure 3). A second approach used slices from the transgenic mice expressing FLAG-tagged D2 receptors (Supplementary Figure 1B)^22^. Brain slices containing the SN from animals expressing FLAG-tagged D2 receptors were incubated in the anti-FLAG M1 antibody conjugated with AF594 for 30 minutes. Cells were identified and imaged at baseline, following 10 minutes of agonist exposure, and following a 30-minute wash period after the agonist (Fig. 6A top row). Similar to the GFP-D2, little receptor internalization was detected following the agonist exposure (either quinpirole or dopamine) or wash period (Fig. 6B). There was no difference in ΔF_r_/F_r_ between neurons expressing FLAG-D2 or the GFP-D2 receptors when using the selective D2 agonist quinpirole or the endogenous D2 agonist dopamine (Fig. 6C).

One advantage of using the M1 anti-FLAG antibody is that the affinity of the antibody is greatly reduced in calcium free solutions. This allows the stripping of antibody from receptors that are located on the plasma membrane^27^. Thus, the application of a calcium-free solution ( + EGTA 100 µM) was used to determine the presence of intracellular FLAG-D2 receptors located near the plasma membrane. When slices were treated with a calcium-free solution for 15 min, the fluorescence of the control group decreased by 61 ± 4% (N = 5; data not shown). When slices were first treated with agonist (quinpirole or dopamine) and washed for 15 min with a calcium-free solution there was a 59 ± 4% decrease in fluorescence (N = 14; data not shown). Thus, there was no agonist-induced change in the plasma membrane-associated FLAG-D2 receptors.

The combined results from the GFP-D2 and FLAG-D2 experiments suggest that D2 receptors are not internalized with prolonged agonist activation. To examine this hypothesis, brain slices containing the locus coeruleus (LC) from animals expressing the FLAG-D2 were imaged. Normally D2 receptors are not expressed on LC cells, but the FLAG-D2 receptors are because the TH promotor drives the expression. Following the application of quinpirole (10 µM) or dopamine (100 µM), the FLAG-D2 receptors were internalized significantly more than SN neurons. Internalization continued beyond the 10-min application, increasing substantially during the 30-minute wash period (Fig. 6A,B).

### Dopamine neurons internalize FLAG-MORs

Given that D2 receptors were internalized in LC neurons, perhaps dopamine neurons in the SNc lack the requisite machinery for classic receptor internalization. To investigate this, a transgenic mouse model was used that expressed a FLAG-tagged µ-opioid receptor (FLAG-MOR) driven by the TH promotor^27^. This animal therefore expressed FLAG-MORs on dopamine neurons in the SN (Fig. 6D, top row). Following the application of [Met]^5^enkephalin (ME, 30 µM, 10 min) substantial internalization of the FLAG-MOR was observed (Fig. 6D, E). During the 30-minute wash period, a portion of the receptors recycled back to the plasma membrane, although much of the internalization remained. The internalization of FLAG-MOR mimicked what has been previously shown for FLAG-MOR in LC cells^27^ and was replicated here. FLAG-MORs were internalized in LC neurons during agonist exposure followed by slow recovery of the receptor to the membrane (Fig. 6D lower row, 6E). Average values for internalization following 10 minutes of agonist exposure are shown for all groups in Fig. 6F. D2 receptors in dopamine neurons in the SN internalize significantly less than D2 receptors in LC neurons, MORs in SN dopamine neurons, and MORs in LC neurons.

## Discussion

D2 receptors expressed on midbrain dopamine neurons play a critical role in determining the excitability of these neurons. However, anatomically identifying somatodendritic release sites and D2 receptor-containing synapses has proven difficult. Thus, anatomical results suggest that dopamine must travel some distance before finding a D2 receptor^25, 26^. However, functional estimates of the concentration of dopamine at the post-synaptic receptor approach 100 µM^30^ and the distance from release to receptor was estimated to be less than 400 nm^23^. In this study, using the GFP-D2 animal model, receptors expressed at lower levels than wild type were visualized in live neurons. The distribution of D2 receptors was found to be distinctly punctate indicating that the action of dopamine in the soma/dendritic area is localized. This distribution is suggestive of synaptic receptor localization in a molecular complex. Combined with the functional results, these observations suggest that dendrodendritic transmission is more point-to-point than previously proposed.

The present results do not entirely exclude the possibility that D2 receptors can be located on dendrites with no corresponding release site and mediate paracrine-like transmission. Indeed, many electron micrograph studies have identified such receptors^25, 26, 31^. It is possible that there are D2 receptors that do internalize upon desensitization are from this “extrasynaptic” population of receptors while other D2 receptors mediate point-to-point transmission and are much less mobile. It is also possible that the present study underestimates the number of extrasynaptic receptors. These receptors would likely be located diffusely on the membrane and thus the antibodies used would also be diffuse and more difficult to detect within a slice.

One concern in this study was antibody-induced alteration in the distribution of receptors and/or clustering of receptors for the method of live imaging. The use of an anti-GFP nanobody (Supplementary Figure 2) minimized this concern because the same punctate distribution was observed with this method that eliminates antibody crosslinking. In addition, the functional comparison of antibody bound and naked GFP-D2 receptors revealed no differences in signaling. Finally, results from the GFP-tagged and FLAG-tagged D2 receptors are similar with the use of confocal imaging^28^ and live imaging. The two versions of tagged receptors have different modes of expression (knocked-in driven from the endogenous promotor vs. transgenic driven from the TH promotor) and different antibodies are used for these two models (rabbit polyclonal anti-GFP vs. mouse monoclonal anti-FLAG M1) minimizing the possibility that the present results are an artifact of the technique. Additionally, the ability to visualize FLAG-D2 and MOR receptors in multiple neuron types (Fig. 6) supports the specificity of the finding that D2 receptors in their endogenous midbrain neurons desensitize without significant internalization.

Desensitization, or a reduction in signaling during continued or repeated activation with agonist, is one early adaptation that is believed to occur in all GPCRs. Classically, activated G proteins recruit GPCR kinases (GRKs), which phosphorylate the activated GPCR. This, in turn, increases the binding affinity of the intracellular domains of the receptor for arrestin proteins. Arrestins prevent the receptor from further activating G proteins despite being agonist-bound (desensitization). Arrestins also promote internalization of receptors through interactions with clathrin and its adapter protein AP2 (reviewed for example in^32^). The present results indicate that the desensitization of D2 receptors in the SNc occurs largely independent of internalization.

D2 receptor desensitization and internalization have been studied extensively in heterologous expression systems and differ from the classical model in several respects. The D2 receptor undergoes GRK-dependent phosphorylation following the application of agonists^33, 34^ and internalization is GRK-dependent in CHO and HEK293 cells. A number of sites are phosphorylated by GRK, but mutation of those sites did not alter function, desensitization, arrestin recruitment, or internalization^35^. Further, GRKs were found to regulate internalization and decrease coupling of the D2 receptor with G proteins independent of receptor phosphorylation^36^. Interestingly, GRKs have been found to co-immunoprecipitate as part of a larger protein complex with the D2 receptor independent of D2 receptor activation^36^. While cell type specific GRK expression data is incomplete, most cell types express multiple GRKs^37^ and isoforms 2, 3, and 5 have been most often studied in relation to D2 receptor regulation. Thus, GRKs are involved in desensitization and internalization of the D2 receptor in heterologous expression systems, but likely due to a scaffolding action or phosphorylation of proteins other than the D2 receptor.

In oocytes, arrestin caused a decoupling of the D2 receptor from G protein signaling in a GRK-independent manner^38^. A sequence of 4 residues in the third intracellular loop of the D2 receptor was identified that when mutated abolished the receptor/arrestin interaction and internalization^39^. When 3 of the 4 residues were mutated, the receptor/arrestin interaction was maintained, but there was no internalization^40^. These findings suggest arrestin is required for desensitization, but indicate that arrestin binding and internalization are dissociable events.

The G protein isoform associated with D2 receptor signal could also be important for desensitization and internalization phenotypes. Expression of D2 receptors in HEK cells upregulated the β5 isoform of G proteins. The association between D2 and G-β5 prevented receptor internalization but not arrestin recruitment^41^. Regulators of G protein signaling (RGS) proteins could also play a role in the desensitization of D2 receptors without internalization. Co-expression of an RGS protein (9-2) in HEK cells prevented internalization of the D2 receptor but not delta opioid receptor^42^.

Thus, the regulation of D2 receptors appears to differ from many other GPCRs. The majority of studies on D2 receptor desensitization and internalization have occurred in heterologous expression systems, whereas this study used live dopamine neurons to investigate these phenomena. The lack of internalization of D2 receptors found in the present study was cell-specific and likely dependent on the distribution and scaffolding of the receptor and the proteins in dopamine neurons that respond and contribute to D2 signaling. This would account for the lack of D2 receptor internalization in dopamine neurons versus the internalization observed in LC neurons. There are very few studies that have imaged the trafficking of tagged membrane-bound receptors on neurons maintained in brain slices. In cultured hippocampal neurons and superior cervical ganglion cells the trafficking of GABA_B_^43^ and alpha-2 adrenergic receptors^44^ were examined, respectively. In each case the receptors were evenly distributed along the plasma membrane and were internalized with the application of a high agonist concentration. To our knowledge, the only place where receptor trafficking has been examined in living brain slices are FLAG-tagged µ-opioid receptors in the LC^27^. The present study presents evidence from two distinct constructs of tagged D2 receptors in dopamine neurons demonstrating a distinct distribution and resistance to agonist-induced trafficking that have not been reported elsewhere.

The idea that the D2 receptor resides within a complex of proteins on the cell membrane is an attractive explanation for the punctate appearance seen with the GFP-D2 (Fig. 1). The protein complex could act to anchor the D2 in specific synaptic locations insuring that signaling is rapid and repeatable, and also prevent extensive internalization and recycling of the receptor. In HEK 293 cells, the D2 receptor uniquely localizes to detergent-insoluble membrane fractions resulting in a compartmentalization of the D2 receptor^45, 46^. This suggests that the D2 receptor can reside within dense protein aggregates, a characteristic not seen with opioid receptors in the same studies.

Apart from GRK and arrestin, which proteins could associate with the D2 receptor to regulate desensitization or act as scaffold? The large third intracellular loop of the receptor likely mediates much of the interaction between D2 and other intracellular proteins, many of which are scaffolding proteins. The third intracellular loop contains a binding site for the scaffolding protein spinophilin, which is known to also bind and target to dendritic spines the PP1 protein^47^. Additionally, the D2 receptor has been shown to interact with the actin binding protein APB-280 (a.k.a. filamin-A)^48^, which could aid in determining receptor expression, membrane stability, and localization^49, 50^. The actin cytoskeleton adapter protein 4.1N^51^ and the RGS19 interacting protein GIPC, which contains a PDZ domain and binds to the D2 C-terminus^52^, are additional scaffolding proteins shown to interact directly with the D2 receptor. It is unclear which of these proteins might be responsible for stabilizing the location of D2 receptors in dopamine neurons.

Two primary observations are made in the present study. The punctate and immobile distribution of D2 receptors on midbrain dopamine neurons supports the idea that dendrodendritic neurotransmission between these neurons is at least partially directed at what appear to be synapses. Additionally, receptor desensitization and internalization in live neurons are dissociable specifically for the D2 receptor in dopamine neurons. Thus, the distribution and regulation of D2-autoreceptors are key components of dendro-dendritic communication between dopamine neurons. The present study sacrificed some fluorescence resolution (i.e. culture preparations) for the ability to track native receptors in acute brain slice preparations. Further examining clustering of D2 receptors will require the labeling of receptors with single fluorescent ligands in combination with high resolution imaging in order to determine the number of receptors contained in a single puncta and changes that may occur.

## Methods and Materials

### Animals

All animal procedures and protocols were approved by the Institutional Animal Care and Use Committee at Oregon Health Sciences University (OHSU IACUC, protocol# IP00000160). All experiments were performed in compliance with the appropriate guidelines of the Institutional Animal Care and Use Committees at Oregon Health and Science University.

Both male and female mice were used in this study (25–100 days old). Wild type mice (C57BL/6 J) were obtained from The Jackson Laboratory (Sacramento, CA). FLAG-D2^22^ animals are maintained in-house on a D2-KO background. FLAG-MOR^27^ animals are maintained in-house and are on a C57BL/6 J background. All mice were group housed in standard plastic cages on a 12-hour light/dark cycle with food and water available *ad libitum*.

### Generation of Superecliptic pHluorin tagged D2R knockin mouse (GFP-D2)

A Superecliptic pHluorin (SpH)^53, 54^ with a human prolactin hormone signal sequence was fused to the amino terminus of the mouse D2R using standard cloning techniques. The knockin targeting vector consisted of the endogenous D2R from exon 2 with the SpH fused in frame to the D2R amino terminus and a pgkNeoLoxP cloned into the intron between exon 2 and 3 (Fig. 1). The knock-in mice (termed GFP-D2) were generated at the Transgenic Mouse Facility at the University of California at Irvine. The targeting construct was electroporated into JM8.N4 ES cells derived from C57BL/6 NTac mice and positive clones selected by G418 selection. Selected clones were screened by Southern blot and positive clones were microinjected into C57BL/6 mouse blastocytes. Chimeric mice were confirmed by Southern blot. The GFP-D2 mouse line was established by crossing heterozygotes to derive a homozygote line. Genotyping was done by PCR from tail clips using primer pairs, which had one oligo targeted to the flanking region outside of the targeting vector sequence on both the 5′ and 3′ ends to insure homologous recombination had occurred. To excise the pgkNeoLoxP cassette the homozygote mice were crossed with the Cre deleter line C57BL/6N-HPRT^tm1(CMV-cre)Brd/^Mmucd from the Mutant Mouse Regional Resource Center at the University of California at Davis. In this construct the mRNA for the SpH D2 will be transcribed from the endogenous D2 locus. During post translational processing the signal sequence will be cleaved resulting in the D2R protein with the SpH tag on the amino terminus. Since the mice were generated on a C57BL/6 background no back crossing was necessary and C57BL/6 J wild type mice were used as controls.

### Slice preparation and electrophysiology

Isoflurane was used to deeply anesthetize mice prior to decapitation. Brains were rapidly removed and placed in modified Krebs buffer containing (in mM) 126 NaCl, 2.5 KCl, 1.2 MgCl_2_, 2.4 CaCl_2_, 1.4 NaH_2_PO_4_, 25 NaHCO_3_, 11 D-glucose, and with 10 µM MK-801. Horizontal slices (222 µm) were taken using a Leica vibratome and allowed to recover for at least 30 minutes prior to use in Krebs with MK-801 being continuously bubbled with 95/5% O_2_/CO_2_. Slicing and recovery was done at elevated temperatures (30–34 °C). Following recovery, slices were secured in a recording chamber maintained at 32–35 °C and perfused with modified Krebs at a rate of 3 ml/min. Whole-cell voltage clamp electrophysiological recordings were made from dopamine neurons in the SNc identified by a location lateral to the medial terminal nucleus of the accessory optic tract. Dopamine neurons were identified by electrophysiological characteristics measured in cell-attached mode (spontaneous pacemaker action potential firing 1–5 Hz, wide action potential width ~ 2 ms)^55^, characteristic cell properties such as capacitance and resting membrane resistance^56^, and by the presence of a slow I_h_ current induced by hyperpolarization. Two internal recording solutions were used; EGTA internal with (in mM) 0.1 EGTA, 130 K-methanesulphonate, 20 NaCl, 1.5 MgCl2, 10 HEPES (K), 2 ATP, 0.2 GTP, 10 phosphocreatine and BAPTA internal with 10 BAPTA (4 K), 90 K-methanesulphonate, 20 NaCl, 1.5 MgCl2, 10 HEPES (K), 2 ATP, 0.2 GTP, 10 phosphocreatine. Recordings were made with glass electrodes with a tip resistance of 1.3–1.8 MΩ when filled with internal. Immediately following break-in, cell capacitance, input resistance, and series resistance was measured by an average of 10 applied pulses ( ± 5 mV for 50 ms sampled at 50 kHz and filtered at 10 kHz) computed in Axograph. Throughout the experiment series resistance was monitored to ensure recording stability and recordings were discarded if series resistance exceeded 12 MΩ. In all recordings, internal solution was dialyzed for at least 10 minutes prior to the onset of experimental protocol. All drugs and compounds were bath applied. During the desensitization protocol, a slice was exposed to only a single saturating concentration of one dopamine D2 receptor agonist (quinpirole or dopamine) and the GABA_B_ agonist (baclofen) then discarded. Data for bath application experiments was acquired with Chart 7 (AD instruments, Colorado Springs, CO). Experiments using electrical IPSC stimulation were conducted in modified Krebs with 100 µM picrotoxin, 10 µM DNQX, and 300 nM CGP55845. For stimulated IPSC generation, a glass electrode was inserted into the slice ~20 µM away from the cell to act as a monopolar stimulating electrode. Once per minute, five 0.5 ms pulses were applied to the slice at 40 Hz and recorded using AxoGraph X software (Berkeley, CA). The amplitude and kinetics of the IPSCs were determined using AxoGraph software from an average of at least five IPSCs. Recording quality and access resistance during these experiments was monitored by applying a brief (500 ms) hyperpolarizing (to −70 mV) step prior to each IPSC stimulation.

In some experiments, exogenous dopamine was applied to the cell via LED photolysis of a caged dopamine compound (see below for synthesis information). This compound was dissolved in modified Krebs at a concentration of 100 µM and continually recirculated over the slice. A LED (365 nm) was flashed once per minute and the results acquired using ScanImage software^57^. Flashes of 10, 50, and 100 ms duration were used on every cell tested and amplitudes and kinetics were determined from an average of at least 3 flash-induced currents using AxoGraph software. No noticeable rundown of the caged dopamine compound occurred during experiments of ~30 minutes.

### Imaging of live slices

The Superecliptic pHluorin was not visible in live brain slices using 2-photon activation or in fixed brain slices using confocal microscopy. However, because the SpH is a derivative of GFP, incubating either fixed or live brain slices in an anti-GFP antibody reliably amplified the signal so as to be readily visualized with either method. For live imaging, slices were incubated in AF594-conjugated anti-GFP (GFP-D2, 1:400 in modified Krebs) or AF594-conjugated M1 antibody (FLAG-D2 and MOR) for 20–30 minutes before being mounted in a recording chamber. Slices were observed using a custom-built 2-photon microscope with ScanImage software^57^. Simultaneous viewing with a scanning DIC allowed visualization of the slice/cells and the fluorescence. Z-stacks (~20 images, 1 every micron) of identified cells were acquired every 10 minutes before, during, and after agonist exposure. For some experiments, cells with substantial antibody staining were identified then patched with a recording pipette containing BAPTA internal with AF488 and dialyzed for at least 10 minutes prior to imaging. All image processing and analysis was done using FIJI. For internalization analysis, 4–5 images (out of the z-stack) in the diametric center of the cell body were summed for each time point. For each summed image, fluorescence measurements were taken from areas with no clear antibody staining (background) and all other measurements were normalized to the image background. To measure membrane-bound and internal fluorescence, a ROI was carefully drawn just outside of the membrane of the whole soma and a fluorescence measurement taken. Then a ROI was carefully drawn just inside of the membrane around the whole soma and a fluorescence measurement taken. This second ROI served as the internalized fluorescence and was subtracted from the first ROI to get the membrane fluorescence. The ROIs were drawn on the first image (baseline) and copied onto the images for the remainder of the time points to ensure the measurements were equivalent. The ratio of internal to membrane fluorescence (F_r_) was the most reliable measurement of internalization.

### Anti-GFP nanobody expression and purification

A nanobody recognizing GFP was obtained from Addgene (Cambridge, MA) and cloned into the pET-22b vector with a C-terminal 8xHis-tag preceded by a thrombin cleavage site. The lysine at 116 of nanobody was mutated to cysteine for a single dye-labeling site. Protein expression was conducted in *E-coli* strain BL21 (New England BioLabs, Ipswich, MA) in terrific broth medium. The cell culture was grown to OD_600_ 0.7 to 1.0 at 37 °C, and protein synthesis was induced by 0.5 mM of isopropyl b-D-1-thiogalactopyranoside and fermentation was carried out at 20 °C for 18 h. Cells were harvested by centrifugation and then lysed in a lysing buffer using a sonicator. The debris was eliminated by centrifugation and the clear supernatant was purified using the HisTrap column (GE Healthcare, Marlborough, PA). The His-tag was removed by adding thrombin protease in to the protein solution at 1:100 (by mass) and incubated at 4 °C overnight. The protein was further purified by size-exclusive chromatography (Superdex 200) using a FPLC system. The protein was a single band by SDS-Page (10–20% gradient) electrophoresis.

### Anti-GFP nanobody AF594 conjugation

A solution containing nanobody (100 µg) was used for the conjugation reaction. The solution was mixed with tris-(2-carboxyethyl)phosphine on ice for 10 min followed with 1.5-fold of AF594 maleimide (2 µl of 5 µg/µl in dimethylsulfoxide). The reaction proceeded on ice for 1 h. The excess dye was removed by P6 spin-column (BioRad, Hercules, CA). The protein-dye ratio was determined by measuring OD at 280 and 594 and was close to 1.

### Immunochemistry and Confocal imaging

Slices were taken and incubated in AF594-conjugated anti-GFP for 30 minutes as above. Slices were then incubated 3x in antibody free Krebs and fixed using 4% PFA in PBS buffer for 2 hours. Permeabilization and blocking were done for 2 hours using 0.2% Triton-X and 0.5% fish skin gelatin. Slices were incubated in rabbit anti-tyrosine hydroxylase antibody (1:1000 in PBS + 0.05% NaN_3_) overnight. Slices were washed in PBS and then incubated in AF488-conjugated goat anti-rabbit antibody for 2 hours. Slices were washed and mounted on #1.5 glass coverslips using an aqueous Fluoromount. Images were collected using a Zeiss confocal LSM 780 confocal microscope with a 20x objective.

### Radioligand Binding

Ventral midbrains were dissected and pooled from two mice for each sample. Samples were homogenized with a PT 1200 E polytron (Kinematica, Inc.) for 10 s on ice in 4 ml Tris buffer (50 mM Tris-HCl, 0.9% NaCl, pH 7.4 at 4 °C) and centrifuged at 30,000 *g* for 20 min. The pellets were resuspended in 4 ml Tris buffer, incubated for 30 min at 25 °C to release endogenous dopamine, centrifuged, and resuspended in Tris buffer. Membranes (37–68 μg of protein) were incubated in duplicate in a total reaction volume of 1 ml with 50 mM Tris containing 0.9% NaCl and 0.002% bovine serum albumin, pH 7.4, and [^3^H]YM-09151–2 (84.4 Ci/mmol; PerkinElmer) at 6 concentrations from approximately 0.01–0.3 nM. Nonspecific binding was determined in the presence of 1 μM spiperone. Reactions were incubated at 25 °C for 1 h and terminated by filtration through 0.05% polyethylenimine-presoaked Wallac Filtermat A filters (PerkinElmer) using a 96-well Tomtec cell harvester and ice-cold wash buffer (10 mM Tris-HCl, pH 7.4 at 4 °C, and 0.9% NaCl). Filters were allowed to dry at least 1 h before adding scintillation fluid to each filtered spot. Radioactivity on the filters was determined using a Wallac 1450 microBeta scintillation counter. Proteins were measured using the BCA method (Pierce Biotechnology).

### Synthesis of caged dopamine (carboxynitroveratryl-dopamine, “CNV-dopamine)

All solvents were purchased in septum-sealed bottles stored under an inert atmosphere. All reactions were sealed with septa through which a nitrogen atmosphere was introduced unless otherwise noted. Reactions were conducted in round-bottomed flasks or septum-capped crimp-top vials containing Teflon-coated magnetic stir bars. Reactions were monitored by thin layer chromatography (TLC) on precoated TLC glass plates (silica gel 60 F_254_, 250 μm thickness) or by LC/MS (Phenomenex Kinetex 2.1 mm × 30 mm 2.6 μm C18 column; 5 μL injection; 5–98% MeCN/H_2_O, linear gradient, with constant 0.1% v/v HCO_2_H additive; 6 min run; 0.5 mL/min flow; ESI; positive ion mode). TLC chromatograms were visualized by UV illumination or developed with *p*-anisaldehyde, ceric ammonium molybdate, or KMnO_4_ stain. Reaction products were purified by flash chromatography on an automated purification system using pre-packed silica gel columns or by preparative HPLC (Phenomenex Gemini–NX 30 × 150 mm 5 μm C18 column). Analytical HPLC analysis was performed with an Agilent Eclipse XDB 4.6 × 150 mm 5 μm C18 column under the indicated conditions. NMR spectra were recorded on a 400 MHz spectrometer. ^1^H chemical shifts were referenced to TMS or residual solvent peaks. Data for ^1^H NMR spectra are reported as follows: chemical shift (δ ppm), multiplicity (s = singlet, d = doublet, t = triplet, q = quartet, dd = doublet of doublets, m = multiplet), coupling constant (Hz), integration.

**CNV-dopamine (5/6**, Figure S1)**:** *N*-Teoc-dopamine^29^ (**1**; 150 mg, 0.504 mmol, 1 eq), 2-(trimethylsilyl)ethyl 2-bromo-2-(4,5-dimethoxy-2-nitrophenyl)acetate^58^ (**2**; 233 mg, 0.555 mmol, 1.1 eq), and K_2_CO_3_ (153 mg, 1.11 mmol, 2.2 eq) were combined in DMF (5 mL) and stirred at room temperature for 8 h. The reaction mixture was subsequently diluted with water and extracted with EtOAc (2 × ). The combined organic extracts were washed with water and brine, dried over anhydrous MgSO_4_, filtered, and concentrated *in vacuo*. Flash chromatography on silica gel (0–25% EtOAc/toluene, linear gradient) separated the two regioisomeric, mono-alkylated products (**3** and **4**; The regiochemistry was not definitively assigned). The isomer that eluted first (**3**, higher R_f_) was obtained as 39 mg (12%) of a pale yellow gum. The second band to elute afforded 60 mg (19%) of **4** as a similar yellow gum.

Isomer **3**: ^1^H NMR (CDCl_3_, 400 MHz) δ 7.71 (s, 1 H), 7.29 (s, 1 H), 7.27 (s, 1 H), 6.94 (d, *J* = 8.2 Hz, 1 H), 6.83 (dd, *J* = 8.2, 2.0 Hz, 1 H), 6.72 (d, *J* = 1.9 Hz, 1 H), 6.41 (s, 1 H), 4.56 (s, 1 H), 4.32 – 4.17 (m, 2 H), 4.17–4.07 (m, 2 H), 3.99 (s, 6 H), 3.31 (q, *J* = 6.6 Hz, 2 H), 2.65 (t, *J* = 7.0 Hz, 2 H), 1.03–0.88 (m, 4 H), 0.02 (s, 9 H), −0.02 (s, 9 H); MS (ESI) calcd for C_29_H_44_N_2_O_10_Si_2_Na [M + Na]^+^ 659.2, found 659.2.

Isomer **4**: ^1^H NMR (CDCl_3_, 400 MHz) δ 7.71 (s, 1 H), 7.37 (s, 1 H), 7.28 (s, 1 H), 6.84 (d, *J* = 2.1 Hz, 1 H), 6.80 (d, *J* = 8.2 Hz, 1 H), 6.58 (dd, *J* = 8.2, 2.0 Hz, 1 H), 6.39 (s, 1 H), 4.59 (s, 1 H), 4.33–4.18 (m, 2 H), 4.18–4.07 (m, 2 H), 3.99 (s, 6 H), 3.38 (q, *J* = 6.3 Hz, 2 H), 2.71 (t, *J* = 6.8 Hz, 2 H), 1.02–0.87 (m, 4 H), 0.03 (s, 9 H), −0.02 (s, 9 H); MS (ESI) calcd for C_29_H_44_N_2_O_10_Si_2_Na [M + Na]^+^ 659.2, found 659.2.

Isomers **3** and **4** were then each deprotected with TFA in CH_2_Cl_2_; the following procedure for **3** is representative. Isomer **3** (36 mg, 56.5 μmol) was taken up in CH_2_Cl_2_ (3 mL), and trifluoroacetic acid (0.6 mL) was added. The reaction was stirred at room temperature for 2 h. HPLC/MS analysis of the crude reaction mixture was consistent with complete removal of the Teoc and TMSE protecting groups, with concomitant cyclization of the carboxylic acid and phenol to form a lactone. Toluene (3 mL) was added; the reaction mixture was concentrated to dryness and then azeotroped with MeOH three times. The resulting residue was redissolved in MeOH (2 mL). Aqueous NaOH (2 M, 141 μL, 5 eq) was added, and the reaction was stirred at room temperature for 2 h. HPLC/MS showed complete hydrolysis of the lactone to afford the desired product (CNV-dopamine, **5**/**6**). The solution was neutralized with 2 M HCl (141 μL) and directly purified by reverse phase HPLC (10–50% MeCN/H_2_O, linear gradient, with constant 0.1% v/v TFA additive) to afford one isomer(**5**) of the desired product as a pale yellow solid (15 mg, 52%, TFA salt). Likewise, isomer **4** (57 mg, 89.5 μmol) was deprotected to provide 26 mg (57%, TFA salt) of the other regioisomer of CNV-dopamine(**6**) as a pale yellow solid.

CNV-dopamine, isomer **5**: ^1^H NMR (DMSO-*d*_6_, 400 MHz) δ 7.69 (s, 3 H), 7.66 (s, 1 H), 7.51 (s, 1 H), 6.92 (d, *J* = 1.8 Hz, 1 H), 6.79 (d, *J* = 8.1 Hz, 1 H), 6.73 (dd, *J* = 8.1, 1.9 Hz, 1 H), 6.28 (s, 1 H), 3.89 (s, 3 H), 3.87 (s, 3 H), 3.00–2.85 (m, 2 H), 2.73–2.63 (m, 2 H); Analytical HPLC: t_R_ = 7.8 min, > 99% purity (10–95% MeCN/H_2_O, linear gradient, with constant 0.1% v/v TFA additive; 20 min run; 1 mL/min flow; ESI; positive ion mode; detection at 254 nm); MS (ESI) calcd for C_18_H_21_N_2_O_8_ [M + H]^+^ 393.2, found 393.0.

CNV-dopamine, isomer **6**: ^1^H NMR (DMSO-*d*_6_, 400 MHz) δ 7.72 (s, 3 H), 7.65 (s, 1 H), 7.48 (s, 1 H), 6.95 (d, *J* = 8.3 Hz, 1 H), 6.72 (d, *J* = 2.1 Hz, 1 H), 6.57 (dd, *J* = 8.2, 2.1 Hz, 1 H), 6.26 (s, 1 H), 3.88 (s, 3 H), 3.87 (s, 3 H), 3.03–2.90 (m, 2 H), 2.74–2.65 (m, 2 H); Analytical HPLC: t_R_ = 8.0 min, >99% purity (10–95% MeCN/H_2_O, linear gradient, with constant 0.1% v/v TFA additive; 20 min run; 1 mL/min flow; ESI; positive ion mode; detection at 254 nm); MS (ESI) calcd for C_18_H_21_N_2_O_8_ [M + H]^+^ 393.2, found 393.0.

### Materials

MK-801 was obtained from HelloBio (Princeton, NJ). CGP-55845 was obtained from Tocris Bioscience (Minneapolis, MN). Cocaine hydrochloride was obtained from the National Institute on Drug Abuse, National Institutes of Health (Bethesda, MD). All other drugs were acquired from Sigma-Aldrich (St. Louis, MO). The rabbit anti-GFP Alexa Fluor 594 polyclonal antibody primarily used in this study acquired from Life Technologies, Thermo Fisher Scientific (A21312). Other antibodies were acquired from Thermo Fisher Scientific.

### Statistics

Significant differences between groups were determined by unpaired two-tailed t tests for comparisons with two groups, and by two-way ANOVAs for comparisons with more than two groups. ANOVAs were followed with Bonferroni’s multiple comparisons post hoc tests when significance was found. Values are given as means ± SEM and n = number of cells. Statistical analysis and non-linear regression analysis of radioligand binding were performed using GraphPad Prism 6 (GraphPad Software, Inc., La Jolla, CA).

### Availability of materials

The GFP-D2 knock-in mouse line will be made available through Jackson Laboratories.

## Electronic supplementary material

Suppiementary Information
