# Supplementary material for: Desensitized D2 autoreceptors are resistant to trafficking
Source: Sci Rep. 2017 Jun 29;7:4379. doi: 10.1038/s41598-017-04728-z (PMC5491503; doi:10.1038/s41598-017-04728-z)
Supplement: Supplementary file 1 — Suppiementary Information [file 41598_2017_4728_MOESM1_ESM.pdf]

Supplementary Information

Desensitized D2 autoreceptors are resistant to trafficking

Brooks G. Robinson[1], James R. Bunzow[1], Jonathan B. Grimm[2], Luke D. Lavis[2], Joshua T. Dudman[2], Jennifer Brown[2], Kim A. Neve[3], & John T. Williams[1]

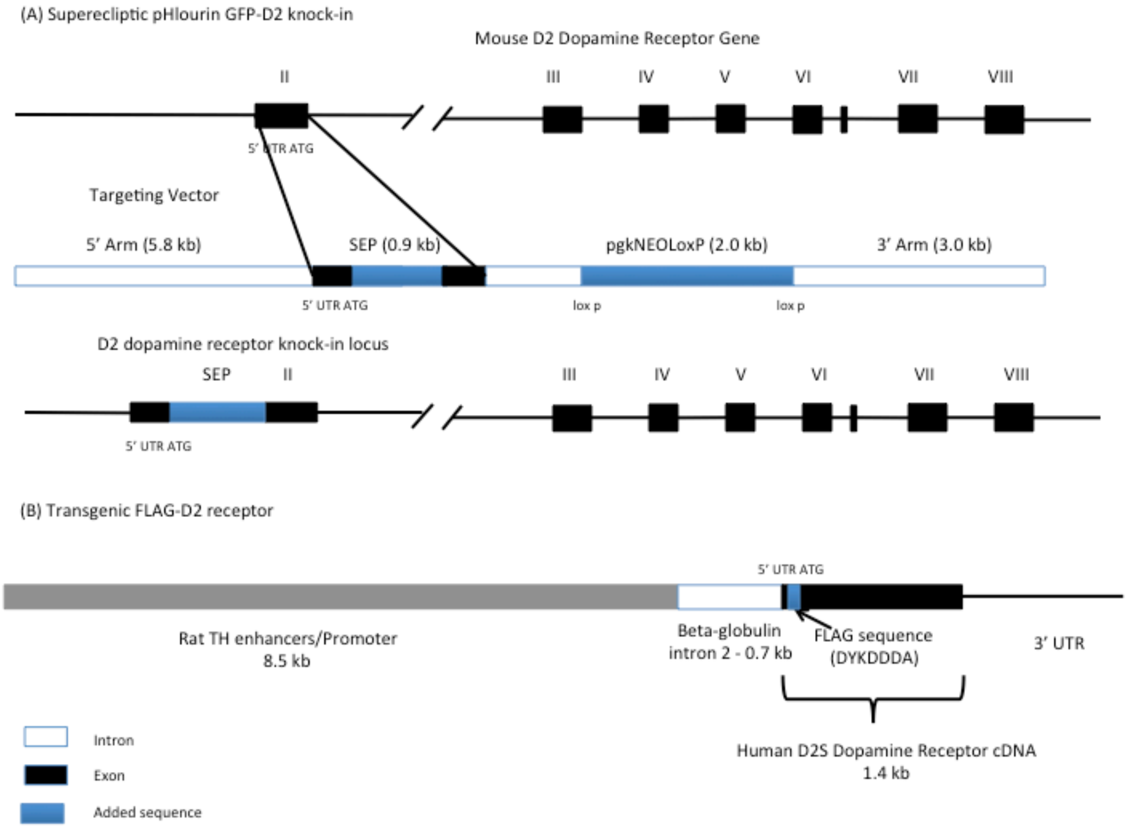

Supplementary Figure S1. Schematic depicting the creation of mouse models expressing tagged D2 receptors. (A) A superecliptic pHluorin (GFP) was inserted into the wild type mouse D2 receptor gene. (B) The FLAG-D2 receptor[22] is the short variant of the human D2 receptor expressed trangenically from the rat TH enhancer/promoter. For both receptors, the exon region preceding the tag consists of a 5' untranslated region and ATG signal. This results in a tag location on the N terminus. See methods for detailed description of GFP-D2 generation.

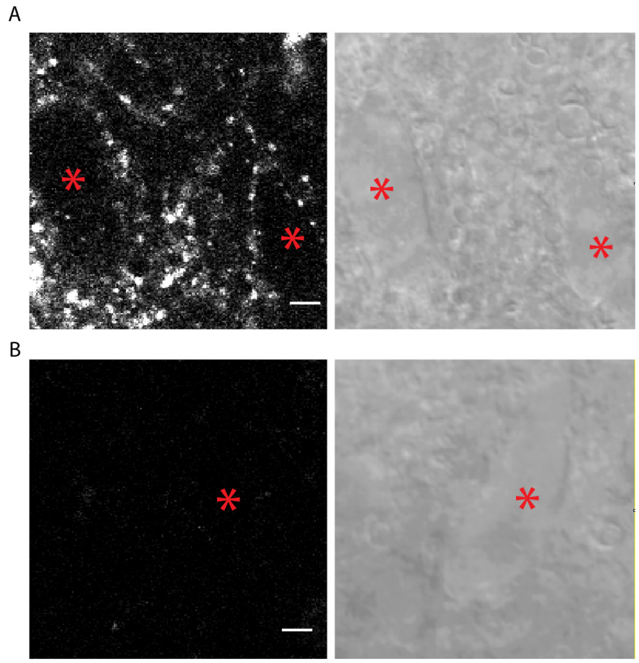

Supplementary Figure S2. Nanobody binding of the GFP-D2 receptor. (A) Simultaneously acquired 2-photon (left) and DIC (right) images of a GFP-D2 slices incubated in the antiGFP AF594 nanobody. Two cells, marked with red asterisks, can be seen with punctate nanobody labeling. (B) 2-photon and DIC images of a WT slice incubated in antiGFP-AF594 nanobody. Very little fluorescence was detected. Scale bars 5 μm.

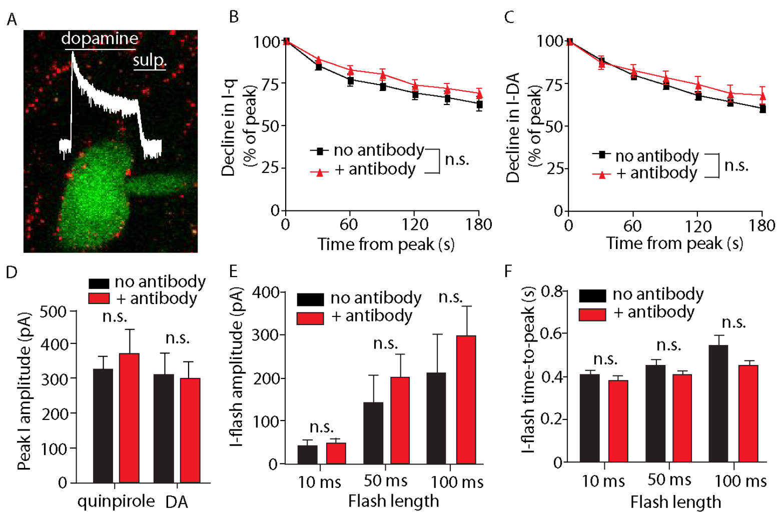

Supplementary Figure S3. Antibody-bound GFP-D2 receptors signal normally (A) Representative image of a neuron being recorded from with BAPTA and AF488 in the pipette. The trace from the recording is shown in white and depicts the outward current induced by bath application of dopamine (100 µM) and the reversal by sulpiride (600 nM). (B, C) Incubation in, and binding of the D2 receptor by the αGFP-AF594 antibody did not alter the decline from peak current induced by the bath application of either quinpirole (10 µM) or dopamine (100 µM) compared to baseline GFP-D2 currents (n=5-7, two-way ANOVA followed by Bonferroni). (D) The αGFP-AF594 antibody did not significantly alter the maximum current induced by quinpirole (10 µM) or dopamine (100 µM) bath application (n=5-7, two-way ANOVA followed by Bonferroni). (E, F) The addition of the αGFP-AF594 antibody to the GFP-D2 receptors did not significantly alter the amplitude or time-to-peak of the uncaged dopamine-induced currents compared to baseline GFP-D2 at any uncaging flash length (n=6, two-way ANOVA followed by Bonferroni). n.s. denotes not significant.
